# Mechanisms and rejuvenation strategies for aged hematopoietic stem cells

**DOI:** 10.1186/s13045-020-00864-8

**Published:** 2020-04-06

**Authors:** Xia Li, Xiangjun Zeng, Yulin Xu, Binsheng Wang, Yanmin Zhao, Xiaoyu Lai, Pengxu Qian, He Huang

**Affiliations:** 1 Bone Marrow Transplantation Center, The First Affiliated Hospital, Zhejiang University School of Medicine, Hangzhou, Zhejiang People’s Republic of China; 2 Institute of Hematology, Zhejiang University, Hangzhou Zhejiang, People’s Republic of China; 3 Zhejiang Engineering Laboratory for Stem Cell and Immunotherapy, Hangzhou, Zhejiang People’s Republic of China

**Keywords:** Hematopoietic stem cells, Aging, Single-cell sequencing, Epigenetics, Rejuvenation

## Abstract

Hematopoietic stem cell (HSC) aging, which is accompanied by reduced self-renewal ability, impaired homing, myeloid-biased differentiation, and other defects in hematopoietic reconstitution function, is a hot topic in stem cell research. Although the number of HSCs increases with age in both mice and humans, the increase cannot compensate for the defects of aged HSCs. Many studies have been performed from various perspectives to illustrate the potential mechanisms of HSC aging; however, the detailed molecular mechanisms remain unclear, blocking further exploration of aged HSC rejuvenation. To determine how aged HSC defects occur, we provide an overview of differences in the hallmarks, signaling pathways, and epigenetics of young and aged HSCs as well as of the bone marrow niche wherein HSCs reside. Notably, we summarize the very recent studies which dissect HSC aging at the single-cell level. Furthermore, we review the promising strategies for rejuvenating aged HSC functions. Considering that the incidence of many hematological malignancies is strongly associated with age, our HSC aging review delineates the association between functional changes and molecular mechanisms and may have significant clinical relevance.

## Background

A key step in hematopoietic stem cell (HSC) aging research was achieved in 1996, revealing that HSCs from old mice were only one-quarter as efficient as those from young mice at homing to and engrafting the bone marrow (BM) of irradiated recipients [1]. This landmark discovery established that the HSC aging process is accompanied by functional decline. Since then, differences between young and aged HSCs have been elucidated from multiple aspects, and the mechanisms of HSC aging have been gradually illustrated. Furthermore, in the clinic, donor age is carefully considered in HSC transplantation, and young donors result in better survival after HSC transplantation [2–4].

Aged HSCs are inferior to young HSCs and show incomplete reconstitution potential. For example, in primary transplantation experiments, compared with young HSCs, aged HSCs showed an overall reduction in long-term repopulating potential [1] and differentiation bias [5]. In the second transplantation experiments, BM cells from old animals were less able to engraft later passage recipients than those from young animals [6]. These findings demonstrated that HSC functions are partially dysregulated during aging and that approaches to rejuvenate aged HSCs should be further elucidated.

Different studies have explored the mechanisms by which aged HSC dysfunction occurs. Altered expression levels of multiple genes and mutation of some specific genes were shown to lead to HSC aging [7]. In addition, inhibition of some signaling pathways, such as the mammalian target of rapamycin (mTOR) and p38 mitogen-activated protein kinase (MAPK) pathways, was closely related to HSC aging [8, 9]. Furthermore, epigenetic perturbations also drove both cellular functional attenuation and other aging manifestations [10]. Finally, some factors in the HSC niche, such as cytokines and enzymes, are also crucial during the aging process [11].

In the present review, we compare the differences in the hallmarks, signaling pathways, and epigenetics of young and aged HSCs, and provide an overview of the BM niche wherein HSCs reside. Notably, we summarize the very recent studies which dissect HSC aging at the single-cell level. In addition, we review the promising strategies for rejuvenating aged HSC functions. Considering that the incidence of many hematological malignancies is strongly associated with age, our HSC aging review delineates the association between functional changes and molecular mechanisms and may have significant clinical relevance.

### Changes in the HSC hallmarks during aging

The functions of HSCs, one of the most important blood cell types, decline in both mice [12] and humans [13] during the aging process. Here, we summarize the changes in the hallmarks of HSC aging with regard to self-renewal, differentiation bias, homing, and engraftment.

#### Self-renewal

HSCs are characterized by their capacity for long-term self-renewal and the ability to generate all functional blood cells. Although different studies demonstrated a dramatic increase in the number of mouse HSCs with age [14, 15], the ability of HSCs to self-renew did not increase accordingly. To further compare young and old HSC self-renewal activity in vivo, Dykstra et al. performed secondary transplantations and found that old HSCs showed less self-renewal activity and generated smaller daughter clones in extended serial transplants than their young counterparts [6]. These phenomena are consistent with the results that most HSCs are actively cycling during fetal life and old age, while HSCs in adulthood are often associated with quiescence [1, 16, 17]. Studies on HSCs in aged mice show an overall decrease in cell cycle activity, with old HSCs undergoing fewer cell divisions than young HSCs [18, 19]. For example, the transition from active cell cycling in fetal HSCs to quiescence in adult HSCs was associated with changes in gene expression programs, including a marked reduction in the expression of *Sox17*, a transcription factor required for the maintenance of fetal but not adult hematopoiesis [20, 21]. The expression levels of other genes associated with the cell cycle, such as Xrcc5, Cdadc1, Cct5, and Polr2h, are also changed during HSC aging [22].

#### Differentiation bias

Compared with young HSCs, aged HSCs have more myeloid differentiation potential and less B cell and T cell output after transplantation into young irradiated recipients. For example, Rossi et al. [23] found a significant reduction in the ability of old long-term HSCs (LT-HSCs) to give rise to peripheral B lymphocytes and a corresponding trend of old LT-HSCs toward increased myelopoiesis. In 2016, Nilsson et al. [24] further found that the levels of common lymphoid progenitors decreased and the frequencies of megakaryocytes and erythrocyte progenitors increased with age. Another important feature in the differentiation of aged HSCs is platelet bias. Grover et al. observed that a very high proportion of aged HSCs almost exclusively produced platelets and that HSC aging was accompanied by a coordinated upregulation of platelet lineage gene expression [25]. The lineage bias during aging was accompanied by the systemic downregulation of genes mediating lymphoid specification and function (e.g., *Bcl11b*, *Blnk*, *Cd160*, *Cd86*, *Csk*) and upregulation of genes involved in specifying myeloid fate and function (e.g., *Amp3*, *Anxa7*, *Ap3b1*, *Arhgef12*, *Cbfa2t1h*) [7, 23, 26]. In humans, the expression levels of some specific genes have been found to exhibit the same change tendencies as those in mice [13, 27]. For example, upregulated genes in aged HSCs, such as *Selp*, specify myeloerythroid fate, while downregulated genes, such as *Flt3* and *Sox4*, are usually associated with lymphopoiesis [13].

#### Homing and engraftment

HSC transplantation is a normal and effective way to assess the functions and potential of HSCs. To test stem cell implantation ability, Liang et al. [28] injected young or old BM cells into congenic mice, and they found that the homing efficiency of old mouse was approximately three-fold lower than that of young mouse. Some specific genes have been demonstrated to be crucial in regulating HSC repopulation, such as *Cdc42*, *Ccr9*, *Gnrh2*, and *Lep* [29, 30]. *CD44* is critical in the maintenance and migration of HSCs [31], and the absence of *CD44* in neonatal BM was shown to enhance the long-term engraftment potential of HSCs. Additionally, p16^Ink4a^, a cyclin-dependent kinase inhibitor, has been shown to play an important role in stem cell regulation and HSC aging [18]. p16^Ink4a^-positive cells accumulate during adulthood, and this accumulation negatively influences lifespan and promotes age-dependent changes in the kidney and heart [32, 33]. Janzen et al. found that p16^Ink4a^ expression in HSCs increased with age and that the absence of p16^Ink4a^ could mitigate the repopulating defects and apoptosis in HSCs [18]. Moreover, *Klf5* is associated with BM homing, and its enrichment is also found in LT-HSCs during aging [34–36].

### Changes in the intrinsic signaling pathways during HSC aging

The functional decline in aged HSCs is also associated with some important signaling pathways. Here, we review the current understanding of the signaling pathways that are differentially activated or repressed during HSC aging, including the DNA damaging, Janus kinase and signal transducer and activator of transcription (JAK/STAT), nuclear factor (NF)-κB, mTOR, transforming growth factor (TGF)-β, Wnt, reactive oxygen species (ROS), and mitochondrial unfolded protein response (UPR^mt^) pathways.

#### DNA damaging pathways

DNA damage is caused by physical, chemical, and biological factors [37] and can block genome replication and transcription. The accumulation of DNA damage during aging has been observed in many studies. Rübe et al. [38] observed an increase in endogenous γH2AX-foci (a sensitive parameter for detecting DNA double-strand breaks) levels in HSCs from elderly donors. Beerman et al. [39] found that age-associated DNA damage accrual was greatest within the HSC compartment among diverse hematopoietic progenitor cells. Genome-wide analysis of young and old HSCs also identified some genes involved in DNA repair that are downregulated with age, such as *Xab2*, *Rad52*, and *Xrcc1* [7]. A specific type of DNA damage is caused by the erosion of telomeres [40], and telomere shortening also occurs during aging [41, 42].

DNA damage leads to a cascade of cellular events known as the DNA damage response (DDR). The DDR is associated with age and is regulated by some important pathways, such as the nucleotide excision repair (NER) and nonhomologous end-joining (NHEJ) pathways. NER plays an important role in maintaining the functional capacity of LT-HSCs during aging by preserving the reconstitution ability, self-renewal potential, and proliferative capacity and by preventing programmed cell death under conditions of stress [43]. The NER pathway-associated gene *Xab2* was shown to be downregulated in aged HSCs [7], suggesting that the NER pathway acts to restore HSC function but is weakened during aging. Another DNA repair pathway is the NHEJ pathway. Nijnik et al. [44] reported that hypomorphic mutations of murine ligase IV (Lig4y288c), a protein implicated in the NHEJ pathway, led to an age-dependent defect in hematopoiesis during aging. In addition, mice deficient in KU70 (a key component of the NHEJ pathway) exhibited severe defects in self-renewal, competitive repopulation, and BM hematopoietic niche occupancy [45]. Consistently, KU70 expression in HSCs was negatively correlated with donor age [46]. Taken together, these observations suggest that the NHEJ pathway may act to preserve HSC functions, and its downregulation during aging may contribute to HSC functional loss.

#### The JAK/STAT, NF-κB, and mTOR pathways

The JAK/STAT signaling pathway is a conserved metazoan signaling system that plays an important role in the immune response, homeostasis, and regenerative processes [47]. Recently, a study using single-cell transcriptomics revealed JAK/STAT signaling functions in stem cell exhaustion during aging [48]. Kirschner et al. showed that approximately 25% of p53-activated old HSCs coexpressed cell cycle inhibitory and proliferative transcripts from JAK/STAT signaling, partially explaining the prolonged cell proliferation, myeloid skewing, and stem cell exhaustion [48].

NF-κB is also known to be an important regulator of HSC aging, and its activity varies at different developmental stages [49]. Stein et al. showed that loss of the NF-κB subunit RelA/p65 severely impaired HSC functions, which occurred in conjunction with increased HSPC cycling, extramedullary hematopoiesis, and differentiation defects [50]. Chambers et al. demonstrated that 71% of 22-month-old HSCs showed enhanced nuclear localization of the p65 protein (an NF-κB subunit), in contrast to only 3% in 2-month-old HSCs, suggesting improved NF-κB activity in aged HSCs [7]. In addition, aged HSCs failed to downregulate *Rad21*/cohesion, a critical mediator of NF-κB signaling [51]. These results suggested that aged HSCs exhibit increased NF-κB activity.

The mTOR pathway regulates cell growth, memory, and aging by receiving signals from mitogenic growth factors, nutrients, and cellular energy levels [52–54]. Chen et al. observed that the levels of phosphorylated (p-)mTOR and mTOR activity were significantly higher in HSCs from aged mice than in those from young mice [8].

#### The TGF-β signaling pathway

The TGF-β pathway plays important roles in regulating HSC behaviors, such as quiescence, self-renewal, and differentiation [55]. Challen et al. showed that exposure to TGF-β1 exerted a stimulatory effect on myeloid-biased HSC proliferation and inhibited the turnover of lymphoid-biased HSCs in young mice [56]. In contrast, a striking reduction in myeloid cell production was found in old mice treated with TGF-β1, and aged HSCs were shown to be more sensitive to TGF-β1 than young HSCs [57]. A genome-wide transcriptome analysis also directly showed that the expression levels of regulatory genes in the TGF-β pathway (such as *Smad4*, *Endoglin*, *Spectrin b2*, *Nr4a1*, *Cepba*, *Jun*, and *Junb*) were reduced during HSC aging, demonstrating that TGF-β signaling in HSCs declines with age [7, 58].

#### The Wnt pathway

Polarity is associated with specialized functions in HSC, such as migration or division, while the loss of polarity has been correlated with reduced self-renewal and altered differentiation of HSC [59, 60]. The small RhoGTPase Cdc42 showed elevated activity in aged HSCs [61] and a correlation with polarity loss in aged HSCs [59]. Further study of the Cdc42 polarity pathway revealed that a shift from canonical to noncanonical Wnt signaling caused HSC aging [62]. Wnt5a treatment of young HSCs activated Cdc42 and induced aging-associated stem cell polarity, a reduction in regenerative capacity, and aging-like myeloid-lymphoid differentiation skewing.

#### The ROS and UPR^mt^ pathway

HSCs reside in a low-oxygen BM niche and maintain low ROS levels [63]. The hypoxic niches are essential for quiescence and protect HSC from apoptosis and loss of self-renewal potential. However, with the accumulation of ROS levels, the self-renewal capacity and repopulation ability of HSC decline during aging process [64]. The thioredoxin-interacting protein (TXNIP) is a regulator of p53 and plays a pivotal role in the maintenance of the hematopoietic cells by regulating intracellular ROS during oxidative stress [65]. TXNIP-p38 axis also acts as a regulatory mechanism in HSC aging by causing lineage skewing, a decrease in engraftment, and an increase in ROS [66]. In addition, Forkhead O (FoxO) proteins play essential roles in the response to physiologic oxidative stress and thereby mediate quiescence and enhance survival in the HSC compartment [67].

Mitochondrial stress generates a reactive oxygen species (ROS)-dependent retrograde signal that modulates neutral stem cell proliferation and differentiation [68]. The *UPR*^*mt*^ is an emerging adaptive stress response pathway that ensures optimal quality and function of the mitochondrial proteome [69]. The expression of a UPR^mt^ regulator, *Sirt7*, was found to be reduced in aged HSCs, and its inactivation compromised the regenerative capacity of HSCs; furthermore, *Sirt7* upregulation improved the regenerative capacity of aged HSCs [70]. Moreover, treatment with the UPR^mt^ stimulator nicotinamide riboside induced the synthesis of prohibition proteins and rejuvenated muscle stem cells in aged mice [71].

**Fig. 1:**
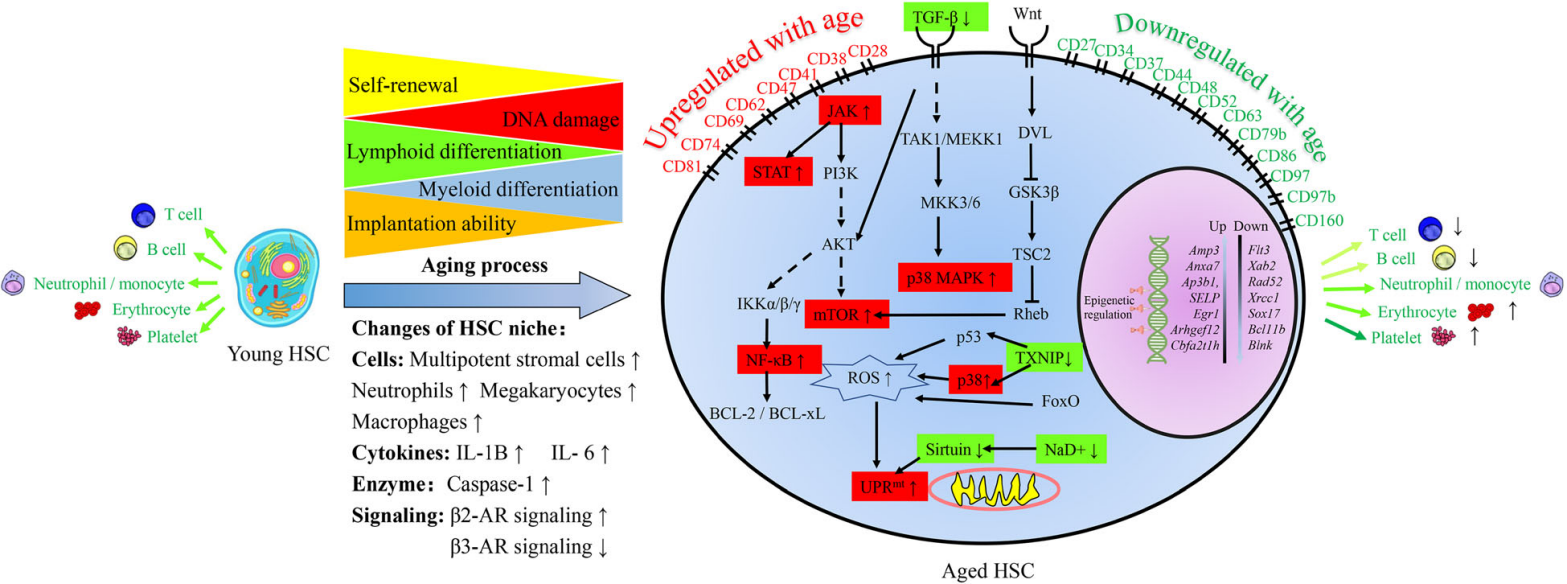
Functional alterations and HSC aging mechanisms. Aging negatively affects HSC functions, including decreasing self-renewal ability and myeloid/platelet-biased differentiation and impairing implantation ability. The intrinsic mechanisms are illustrated at the gene level, signaling pathway level, and epigenetic level. HSC aging is accompanied by some cell surface markers being upregulated with age (such as CD28, CD38, CD41, CD47, CD62, CD 69, CD74, and CD81) and some being downregulated with age (such as CD27, CD34, CD37, CD44, CD48, CD52, CD63, CD79b, CD86, CD97, CD97b, and CD160). Furthermore, aged HSCs show different expression levels of specific genes. For example, *Amp3*, *Anxa7*, *Ap3b1*, *SELP*, *Egr1*, *Arhgef12*, and *Cbfa2t1h* are upregulated, and *Flt3*, *Xab2*, *Rad52*, *Xrcc1*, *Sox17*, *Bcl11b*, and *Blnk* are downregulated. In addition, some signaling pathways are activated/repressed during HSC aging, including the JAK/STAT-NF-κB-mTOR pathway, TGF-β pathway, Wnt pathway, and ROS and UPR^mt^ pathway. Age-related epigenetic regulation includes DNMT1, DNMT3A, DNMT3B, H3K4me3, and H3K27me3. Extrinsic mechanisms include HSC-surrounding cells (including MSCs, neutrophils, megakaryocytes, and macrophages), cytokines (including IL-6 and IL-1B), enzymes (including caspase-1), and β-adrenergic nerve signaling (including increased β2-AR signaling and decreased β3-AR signaling). The red box indicates that the molecule is upregulated with age, and the green box indicates that the molecule is downregulated with age

In summary, these studies demonstrate that signaling pathways can form a network of metabolic processes and provide a systematic way to explain alterations in HSCs during aging (Fig. 1). Future studies should focus on the crosstalk among different signaling pathways and how pathways act synergistically and antagonistically with each other during HSC aging.

### Changes at the intrinsic epigenetic level during HSC aging

Epigenetics refers to changes in gene expression but does not involve changes in the DNA sequence of organisms. Loss of epigenetic regulation at the chromatin level may drive both cellular functional attenuation and other manifestations during aging [72]. In this section, we compare the differences in the DNA methylation, histone modification, and noncoding RNAs in young and old HSCs. In addition, we summarize the research on epigenetic modification by using single-cell epigenetic technologies.

#### DNA methylation

DNA methylation has been shown to increase during the HSC aging process [58]. Beerman et al. [10] showed that DNA methylation changes during HSC aging occur in regions associated with HSC proliferation and lineage-biased differentiation. Furthermore, genome-wide epigenetic and transcriptome profiling identified some important age-associated regulators of methylation, such as DNA methyltransferase 1 (DNMT1), DNMT3A/B, and TET1/2 [15, 73].

DNMT1 has been shown to be essential for HSC self-renewal [74], and loss of DNMT1 causes myeloid skewing [75, 76]. Challen et al. demonstrated that conditional knockout of DNMT3A in HSCs led to increased self-renewal at the expense of differentiation [77]. Another DNMT family member, DNMT3B, was also found to be essential for HSC differentiation [77]. TET2 has also been reported to regulate HSC differentiation and increase myeloid output [78, 79]. In addition, 5-methylcytosine (5-mC) and 5-hydroxymethylcytosine (5-hmC) are two important epigenetic modifications during HSC aging [80]. The loss of the mean 5-mC content in aged leukocytes is 2% of that in young adult leukocytes [81]. Sun et al. showed a decrease in the 5-hmC level in aged mouse HSCs [58], and Busque et al. found the same phenomenon in human peripheral blood cells [82]. These alterations in aged HSC epigenetics are summarized in Table 1.

**Table 1:** Differences in DNA methylation and histone modification levels between young and aged HSCs

	Alterations with age	Functions	Author and year
**DNA methylation**			
DNMT1	Downregulated	Myeloid skewing and self-renewal defects	Beerman et al. 2013 [10] Sun et al. 2014 [58] Trowbridge et al. 2009 [76] Broske et al. 2009 [75]
DNMT3A	Downregulated	Lead to an increase in self-renewal with age at the expense of differentiation	Beerman et al. 2013 [10] Sun et al. 2014 [58] Challen et al. 2014 [77]
DNMT3B	Downregulated	Lead to an even more severe arrest of HSC differentiation	Sun et al. 2014 [58] Challen et al. 2014 [77]
TET1	Downregulated	Enhance HSC self-renewal; increase B cell production; develop B cell malignancies	Sun et al. 2014 [58] Cimmino et al. 2015 [73]
TET2	Downregulated	Attenuate differentiation and lead to myeloid transformation and myeloid malignancies	Busque et al. 2012 [82] Ko et al. 2011 [78]
5-mC	Not studied	Hypermethylation at promoters associated with lineage potential	Beerman et al. 2013 [10] Oshima et al. 2014 [80]
	Not studied	Hypermethylation selectively targeting PRC2 and PU.1-binding sites	Beerman et al. 2013 [10] Sun et al. 2014 [58] Oshima et al. 2014 [80]
	Not studied	Hypomethylation at the HSC fingerprint genes and rRNA genes	Busque et al. 2012 [82] Oshima et al. 2014 [80]
5-hmC	Downregulated	Not studied	Sun et al. 2014 [58]
**Histone modification**			
H3K4me3	Upregulated	Alter promoter usage and upregulate some genes (*Selp*, *Nupr1*, and *Sdpr*)	Sun et al. 2014 [58]
H3K27me3	Upregulated	Alter promoter usage and downregulate *Flt3* expression with age	Sun et al. 2014 [58]
H4K16ac	Downregulated	Downregulate nuclear polarity with age	Florian et al. 2012 [59] Grigoryan et al. 2018 [83]
H3K27ac	Downregulated	Link to leukocyte activation and apoptotic signaling	Grigoryan et al. 2018 [83] Adelman et al. 2019 [15]
H3K9me2	Downregulated	Anchor lamina-associated domains to nuclear lamin A/C	Grigoryan et al. 2018 [83] Towbin et al. 2012 [84]
H3K4me1	Downregulated	Link to myeloid and erythroid differentiation and functions	Adelman et al. 2019 [15]
H3K23ac	Upregulated	Not studied	Cheung et al. 2018 [85]
H2BS14ph	Upregulated	Not studied	Cheung et al. 2018 [85]
H3K9me2	Upregulated	Not studied	Cheung et al. 2018 [85]

#### Histone modifications

Histone modifications include acetylation, methylation, phosphorylation, sumoylation, and ubiquitination and can impact gene expression by altering chromatin structure and affecting the accessibility of the DNA. There is abundant evidence that histone modifications regulate HSC functions, such as self-renewal and differentiation [86–91]. By comparing young and old mouse HSCs, Sun et al. demonstrated that H3K4me3 expression increased with age and showed a strong relationship with age-associated changes in gene expression [58]. Furthermore, broadening of the coverage and intensity of the H3K27me3 signal was observed in aged HSCs. H4K16ac is another age-associated epigenetic marker and can be pharmacologically regulated by the Cdc42 activity-specific inhibitor (CASIN) [83]. Aged LT-HSCs showed an overall lower level of H4K16ac than young LT-HSCs [59]. A recent study also demonstrated that aging was associated with significant reductions in H3K4me1, H3K27ac, and H3K4me3 [15]. Remarkably, some novel age-associated chromatin markers in hematopoietic progenitors, including H3K23ac, H2BS14ph, and H3K9me2, were observed at the single-cell level by highly multiplexed mass cytometry [85, 92]. These alterations in histone modification during HSC aging are summarized in Table 1. The regions in which histone modifications occur are not random but are associated with specific functions. For example, sites with decreased H3K4me1 were linked to genes involved in myeloid and erythroid differentiation and functions, while loss of H3K27ac was linked to genes associated with leukocyte activation, apoptotic signaling, and histone modifications [15].

#### Noncoding RNAs

Noncoding RNAs are RNAs that are not translated into proteins, including transfer RNA, ribosomal RNA, piwi-interacting RNA, microRNA, and long noncoding RNA (lncRNA). Many noncoding RNAs act as regulatory molecules that control gene expression and impact the epigenetic state [93, 94]. Recently, some noncoding RNAs have been demonstrated to play important roles in HSC functions, and their expression levels change with age. Djeghloul et al. observed that the expression of microRNA miR-125b increased with age in human HSCs [95]. These authors also found that inhibition of miR-125 improved the capacity of HSCs from elderly individuals to generate B cells. Another microRNA cluster, the microRNA-132/212 cluster, was upregulated and enriched during HSC aging [96]. Both overexpression and deletion of the above microRNAs led to inappropriate hematopoiesis with age [28]. In addition to miRNAs, some lncRNAs also show differential expression during aging [97].

In general, DNA methylation, histone modifications, and noncoding RNAs play important roles in regulating HSC functions during aging; however, different layers of epigenetic modifications are not independent. Age-associated histone modification changes are accompanied by alterations in DNA methylation. In HSCs, the inability to remove H3K4me1/2 methylation may prevent DNA methylation from repressing the self-renewal program [87]. Furthermore, regions in which H3K36me3 expression decreased in aged HSCs also displayed DNA hypomethylation [58, 98]. However, few studies have examined the relationship between noncoding RNAs and other epigenetic modifications. Therefore, the synergistic and antagonistic effects among different epigenetic modifications should be further elucidated.

### Changes in the extrinsic HSC niche during aging

**Fig. 2:**
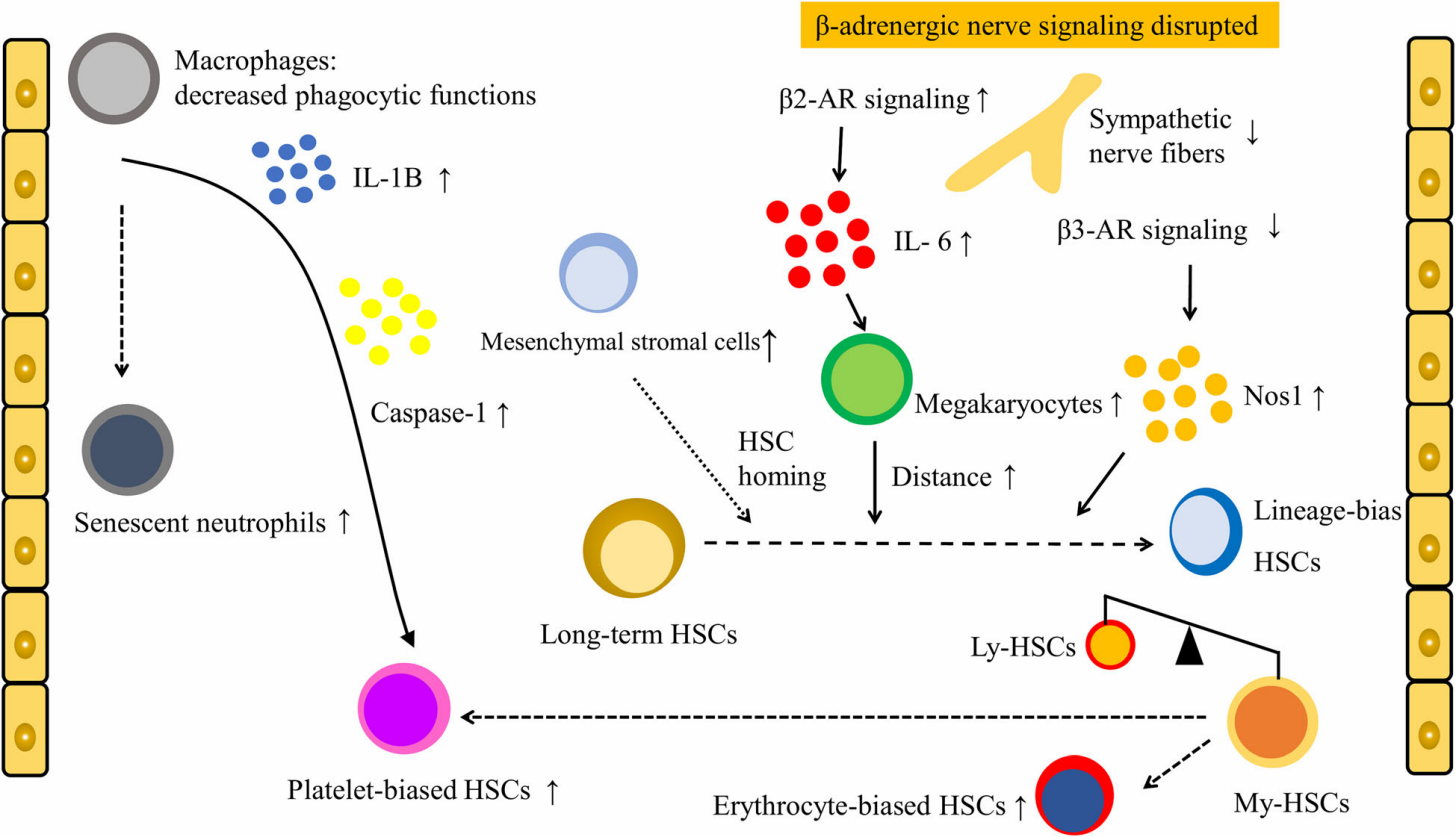
Cell-extrinsic mechanisms of HSC aging involved in HSC-surrounding cells (including megakaryocytes, MSCs, macrophages, and neutrophils), cytokines and enzymes (including IL-6, IL-1B, and caspase-1). Disrupted β-adrenergic nerve signaling (increased β2-AR-IL6-mediated megakaryocyte differentiation and reduced β3-AR-Nos1 activity) is an important determinant of niche alterations during aging, resulting in impaired lymphoid differentiation and myeloid expansion. Dysfunction of aged marrow macrophages directs HSC platelet bias; aged mice have markedly more senescent neutrophils and higher levels of cytokines IL-1B and caspase-1 in their BM niche than young mice. The number of MSCs increases significantly during aging and is associated with replicative senescence and HSC homing

In addition to intrinsic factors, some studies also demonstrated that extrinsic factors affected HSC aging [99, 100]. The BM microenvironment niche is a crucial factor for HSC functions [101]. In the HSC niche, megakaryocytes promote the quiescence of neighboring HSCs [102]. VCAM-1+ macrophages guide the homing of HSPCs to a vascular niche [103]. On the other hand, BM CD169+ macrophages [104] and F4/80+Ly-6G+CD11b+ phagocytic macrophages [105] maintain HSC niches, but their depletion mobilizes HSCs. Furthermore, a recent study found that CD150 high BM Tregs controlled HSC quiescence and engraftment [106]. In addition to the abovementioned hematopoietic cells, nonhematopoietic cells (including mesenchymal stromal cells (MSCs), arteriolar and sinusoidal endothelial cells, and perivascular cells) also play crucial roles in the HSC niche [100, 107, 108]. Moreover, the sympathetic nervous system (SNS) regulates HSC mobilization and orchestrates the release of adrenergic neurotransmitters into the microenvironment in a circadian manner [109–112]. In this section, the effects of HSCs surrounding cells, the SNS, and other factors on HSC aging are summarized (Fig. 2).

In 2018, Maryanovich found that HSC aging critically depended on BM innervation by the SNS around arteriolar niches, as loss of SNS nerves or adrenoreceptor β3 (ADRβ3) signaling in the young mouse BM microenvironment led to premature HSC aging [109]. Furthermore, the distance between HSCs and megakaryocytes regulates HSC proliferation and increases in β3-adrenergic receptor (AR) mice and in natural aging. In 2019, Ho et al. also identified disrupted β-adrenergic nerve signaling (increased β2-AR-interleukin (IL)-6 levels and decreased β3-AR-Nos1 activity) as an important determinant of niche alterations during aging, resulting in impaired lymphoid differentiation and myeloid expansion [113]. In addition, Frisch et al. showed that dysfunction of aged marrow macrophages directed HSC platelet bias and that aged mice exhibited markedly more senescent neutrophils than young mice [11]. Finally, the aged mouse BM niche also expressed elevated levels of cytokines IL-1B and caspase-1. These works highlighted the instructive role of megakaryocytes and macrophages in the age-associated lineage skewing of HSCs.

For nonhematopoietic cells, the number of MSC subsets (such as PDGFRa+SCA1+ and PDGFRa+CD51+ cells) in aged mouse BM was found to be significantly higher than that in young mouse BM [11]. Furthermore, in 2018, Hennrich et al. presented proteome-wide atlases of age-associated alterations in HSPCs, lymphocytes and precursors, monocytes/macrophages and precursors, granulocytes, erythroid precursors, and MSCs [114]. In aged MSCs, prominent alterations included differential regulation of proteins that were associated with cellular responses to stress, replicative senescence, and HSC homing.

### Single cell technologies for HSC aging

#### Single-cell RNA sequencing

Single-cell RNA sequencing has become a powerful tool to characterize distinct functional states at single-cell resolution [115–117]. Several studies using single-cell RNA sequencing have been performed to reveal cell-intrinsic differences during HSC aging [22, 25].

Upon comparing young and old mouse HSCs, Kowalczyk et al. found that cell cycle-related genes dominated the transcriptomic variability and observed fewer cells in the G1 phase among old HSCs [22]. Moreover, old short-term HSCs (ST-HSCs) transcriptionally resembled young LT-HSCs, suggesting that ST-HSCs remained in a less differentiated state. Grover et al. showed that depletion of HSC platelet programming through loss of the *Fog-1* transcription factor was accompanied by increased lymphoid output [25]. Mann et al. found that LT-HSCs from young and aged mice had differential responses to inflammatory challenge and that age-dependent inflammatory myeloid bias was intrinsic to LT-HSCs [34].

In addition to obtaining single-cell transcriptional results from mice, Oetjen et al. also performed a comprehensive assessment of human BM cells using single-cell RNA sequencing [118]. They found consistent increases in chromatin markers in a broad array of cell subtypes from hematopoietic progenitors to terminally differentiated immune cells. Hennrich et al. revealed that the mRNA levels of age-increased glycolytic enzymes were higher in myeloid-primed hematopoietic stem/progenitor cells (HSPCs) than in lymphoid-primed HSPCs, whereas the transcript levels of age-unaffected enzymes were similar in both subsets [114]. These results suggested that the lineage skewing of HSPCs toward myeloid differentiation upon aging was associated with the glycolytic pathway. Adelman et al. observed a decrease in cycling-HSCs and lymphoid-primed multipotent progenitors during aging and identified 364 genes that are differentially expressed with age, such as *Egr1*, *Klf6*, and *Jun* [15]. Overall, these single-cell analyses, summarized in Table 2, helped to demonstrate the intrinsic molecular changes during HSC aging.

**Table 2:** Recent single-cell studies on HSC aging at the genetic and epigenetic levels

Author and year	Age of object Number of analyzed cells /measured cells Cell types	Methods	Conclusion
Kowalczyk et al. 2015 [22]	Young mice: 2–3 months; 176/200 (each type) Aged mice: > 22 months; 176/200 (each type) LT-HSC: LSK CD150+CD48− ST-HSC: LSK CD150−CD48− MPP: LSK CD150−CD48+	SMART-seq	Cell cycle-related genes dominated the transcriptome variability. There was a lower frequency of cells in the G1 phase among old long-term HSCs.
Grover et al. 2016 [25]	Young mice: 2–3 months; 52/61 Aged mice: 20–25 months; 62/74 LSK CD150+CD48−	Fluidigm C1 single-cell AutoPrep system	The principal pathways enriched in young HSCs were involved in cell cycle progression, while those in old HSCs were involved in growth factor signaling.
Mann et al. 2018 [34]	Young mice: 2–3 months; 124~186 (each type) Aged mice: 20–24 months; 124~186 (each type) LT-HSC: LSKCD150+CD48− ST-HSC: LSK CD150−CD48− MPP: LSK CD150−CD48+	SMART-Seq2	LT-HSCs from young and aged mice had differential responses to inflammatory challenge. CD61 was a marker of myeloid-biased LT-HSCs.
Frisch et al. 2019 [11]	Young mice: 1.5–2 months Aged mice: 20–24 months LSK CD150+CD48−Flt3−	Fluidigm C1 single-cell AutoPrep system	Most aged LT-HSCs highly expressed megakaryocyte-biased genes, including *Selp*, *Vwf*, and *Itgb3*. CD41 and CD61 were associated with aged megakaryocytic HSC bias.
Oetjen et al. 2018 [118]	Humans across an age range from 24~84 years 76,645/> 90,000 Mononuclear cells in BM	10X genomics single-cell 3′ solution	The authors identified all the major BM mononuclear populations and age-associated changes in cell population frequencies.
Hennrich et al. 2018 [114]	Young humans: < 30 years; 291 Aged humans: > 50 years; 228 CD34+	SMART-seq2	The mRNA levels of age-increased glycolytic enzymes were higher in myeloid-primed than in lymphoid-primed HSPCs.
Adelman et al. 2019 [15]	Young humans: < 40 years; 338 Aged humans: > 60 years; 310 Lin-CD34+CD38−	Fluidigm C1 single-cell AutoPrep system	The authors observed a decrease in cycling-HSC and lymphoid-primed multipotent progenitors with age.
Florian et al. 2018 [119]	Young mice: 2.5–3 months Aged mice: 20–26 months LSK CD34−Flk2−	scATAC-seq	Young HSCs divided mainly asymmetrically, while aged HSCs divided primarily symmetrically.
Cheung et al. 2018 [85]	Young humans: < 25 years Aged humans: > 65 years Primary human immune cells	Epigenetic landscape profiling using cytometry by time-of-flight (EpiTOF)	The authors found consistent increases in chromatin marks in a broad array of cell subtypes from hematopoietic progenitors to terminally differentiated immune cells.

Single-cell transcriptomics data provide abundant information about the differential gene expression of young and aged HSCs. The expression of surface molecules is a research hotspot, as these molecules can be used to track the aging process of HSCs and assess HSC functions. Using normalized transcription expression matrices, different studies [7, 11, 13, 22, 24, 25, 120, 121] found that CD28, CD38, CD41, CD61, CD47, CD62, CD69, CD74, and CD81 were upregulated with age and that CD27, CD34, CD37, CD44, CD48, CD52, CD63, CD79b, CD86, CD97, CD97b, and CD160 were downregulated with age. Furthermore, some molecules (such as CD9 and CD151) showed contrasting expression tendencies with age in different studies [7, 10, 24]. In addition, a histone 2B-green fluorescent protein label in HSCs (an HSPC-specific GFP label-retaining system) was used to label a reserve stem cell population [122, 123]. These molecules show an exact tendency during aging and could be used to identify aged HSCs and assess HSC functions (Table 3).

**Table 3:** Significant alterations of cell surface markers during HSC aging

Symbol	Alterations with age	Functions
CD9	Upregulated	Adhesion, migration, and platelet activation
CD28	Upregulated	Costimulation
CD38	Upregulated	Cell activation, proliferation, and adhesion
CD41	Upregulated	Platelet activation and aggregation
CD47	Upregulated	Adhesion, activation, apoptosis
CD62	Upregulated	Leukocyte rolling and homing
CD69	Upregulated	Costimulation
CD74	Upregulated	B cell activation
CD81	Upregulated	Activation, costimulation, and differentiation
CD151	Upregulated	Adhesion, signaling
CD27	Downregulated	Costimulation
CD34	Downregulated	Adhesion
CD37	Downregulated	Adhesion, signaling
CD44	Downregulated	Leukocyte rolling, homing, and aggregation
CD48	Downregulated	Adhesion, costimulation
CD52	Downregulated	Costimulation
CD63	Downregulated	Cell motility regulation
CD79b	Downregulated	Subunit of BCR, signaling
CD86	Downregulated	Costimulation of T cells activation and proliferation
CD97	Downregulated	Neutrophil migration, adhesion
CD97b	Downregulated	Neutrophil migration, adhesion
CD151	Downregulated	Adhesion, signaling
CD160	Downregulated	Costimulation

#### Epigenetics at the single-cell level

The development of single-cell epigenomic technologies has allowed the identification of DNA methylation, histone modifications, chromatin accessibility, and chromosome conformation at the single-cell level [117, 124]. For example, single-cell chromatin immunoprecipitation sequencing and single-cell assays for transposase-accessible chromatin using sequencing (scATAC-seq) have been applied to investigate histone modifications and to map accessible chromatin regions.

Florian et al. observed that young HSCs divided mainly asymmetrically, while aged HSCs divided primarily symmetrically [119]. Moreover, the potential of daughter cells was linked to the amount of the epigenetic marker H4K16ac and to the amount of open chromatin. Cheung et al. developed epigenetic landscape profiling using cytometry by time-of-flight (EpiTOF) to measure epigenetic modifications and profile the global levels of a broad array of chromatin modifications in primary human immune cells at the single-cell level [85]. Consistent increases in chromatin markers were found in a broad array of cell subtypes from hematopoietic progenitors to terminally differentiated immune cells, suggesting that systemic changes may result from the reprogrammed chromatin state in hematopoietic progenitors or further upstream in HSCs. These single-cell epigenetic analyses contributed to our understanding of the distinct types of epigenetic alterations occurring during aging at single-cell resolution (Table 2).

### Aged HSC rejuvenation strategies

Currently, there is no doubt that HSCs show declining function during aging, but whether this dysfunction is reversible remains unclear. In 2014, Villeda et al. reported that exposure of an aged animal to young blood can counteract and reverse pre-existing effects of brain aging at the molecular, structural, functional, and cognitive levels, suggesting that some aging-related phenotypes can be improved [125]. In this section, we review the approaches to achieve at least partial rejuvenation of aged HSC functions, including prolonged fasting, genetic modulators, pharmacological intervention, and changing the BM niche (Table 4).

**Table 4:** Aged HSC rejuvenation strategies

Rejuvenation approach	Mechanism	Functions	Author and year
**Prolonged fasting**			
Prolonged fasting	Reduces circulating IGF-1 levels	Promote stress resistance, self-renewal, and lineage-balanced regeneration	Cheng et al. 2014 [126]
**Gene expression regulation**			
*Satb1* overexpression	Epigenetic modification	Restore the lymphopoietic potential of aged HSCs	Satoh et al. 2013 [127]
*Sirt3* overexpression	ROS levels	Restore the long-term competitive repopulation ability	Brown et al. 2013 [128]
*Sirt7* overexpression	Mitochondrial functions	Rescue myeloid-biased differentiation	Mohrin et al. 2015 [70]
**Pharmacological intervention**			
Rapamycin	Inhibition of mTOR	Enhance the regenerative capacity of HSCs from aged mice	Chen et al. 2009 [8]
CASIN	Inhibition of Cdc42	Increase the percentage of polarized cells, restore the spatial distribution of H4K16ac, increase lymphoid output, and reduce myeloid lineage output	Florian et al. 2012 [59]
TN13	Inhibition of p38 MAPK	Decrease ROS level and increase homing ability	Jung et al. 2016 [66]
SB203580	Inhibition of p38 MAPK	Restore the repopulating capacity and maintain quiescence of HSCs	Ito et al. 2006 [9]
ABT263	Inhibition of BCL-2 and BCL-xL	Selectively kill senescent cells	Chang et al. 2016 [129]
**Changing BM niche**			
Engraft into a young niche	Changing the BM niche	Restore the age-related transcriptional profiles of HSCs	Kuribayashi et al. 2019 [130]
Sympathomimetic supplementation	Influencing BM innervation	Improve multilineage cell production and HSC engraftment	Maryanovich et al. 2018 [109]

#### Prolonged fasting

Prolonged fasting reduces progrowth signaling and activates pathways that enhance cellular resistance to toxins in mice and humans [131–133]. Prolonged fasting can protect mice from chemotoxicity by reducing circulating insulin-like growth factor-1 (IGF-1) expression [134, 135]. Notably, Cheng et al. showed that prolonged fasting can rejuvenate HSCs. Prolonged fasting reduces circulating IGF-1 levels and protein kinase A (PKA) activity in various cell populations and promotes stress resistance, self-renewal, and lineage-balanced regeneration [126].

#### Genetic modulators (Satb1, Sirt3, and Sirt7)

HSC aging is accompanied by alterations in gene expression. Therefore, overexpressing some downregulated genes or knocking down the expression of some upregulated genes might be strategies to prevent HSC dysfunction. Reduced *Satb1* expression was found in aged HSCs and associated with compromised lymphopoietic potential, and forced *Satb1* overexpression partially restored that potential [127]. In addition, the expression of *Sirt3*, which regulates the global acetylation landscape of mitochondrial proteins, was suppressed by aging, and *Sirt3* upregulation in aged HSCs improved the HSC regenerative capacity [128]. A similar phenomenon was also found for *Sirt7* [70]. Although overexpression of some regulators has been found to rejuvenate HSCs, few studies have revealed a relationship between gene knockdown and HSC rejuvenation. In the future, gene knockdown might be another credible way to restore HSC functions and identify the functions of some genes in rejuvenation.

#### Pharmacological intervention

##### Rapamycin (mTOR inhibitor)

Aged mice exhibit increased mTOR signaling in HSCs, and rapamycin can enhance the regenerative capacity of HSCs from aged mice, improve their immune response, and extend their life span [8].

##### CASIN (Cdc42 inhibitor)

Cdc42 regulates diverse cellular functions, including cellular transformation, cell division, migration, enzyme activity, and cell polarity [136]. Aged HSCs show elevated Cdc42 activity, and Cdc42 inhibition has been demonstrated to rejuvenate HSC functions. Treated with CASIN in vitro, aged HSCs showed an increase in the percentage of polarized cells, and their H4K16ac level and spatial distribution were rejuvenated to a status similar to that in young HSCs [59]. In addition, CASIN treatment increased the contribution to the B cell compartment in peripheral blood and reduced the contribution to the myeloid lineage. Recently, another study also found that aged HSCs treated with CASIN reestablished an immune system similar to that of young animals [137].

##### TN13 and SB203580 (p38 MAPK inhibitor)

Inhibition of p38 MAPK reduces ROS levels and contributes to HSC rejuvenation. TN13, a cell-penetrating peptide-conjugated peptide, inhibited p38 activity and rejuvenated aged HSCs by reducing ROS [66]. Another inhibitor of p38 MAPK, SB203580, was also found to rescue ROS-induced defects in the repopulating capacity of HSCs and the maintenance of HSC quiescence [9]. These results are consistent with the phenomenon that HSCs reside in a low-oxygen BM niche and that the ROS level of HSCs increases during aging [138, 139].

##### ABT263 (clearance of senescent cells)

One strategy to delay aging is to restore cell functions, while another is to clear senescent cells. Senescent cells accumulate with age and contribute to the development of aging-related diseases [140–142]. Depletion of senescent cells mitigated irradiation-induced premature aging of the hematopoietic system and rejuvenated aged HSCs in normally aged mice. Chang et al. found that ABT263, a specific inhibitor of the antiapoptotic proteins BCL-2 and BCL-xL, selectively killed senescent cells [129]. Oral administration of ABT263 to sublethally irradiated mice and normally aged mice effectively depleted senescent cells, including senescent BM HSCs.

#### Changing the BM niche

Kuribayashi et al. showed that engrafting aged HSCs into young niches restored the age-related transcriptional profiles of HSCs. They transplanted 20-month-old aged HSCs into 10-week-old young mice and later collected aged HSCs engrafted in young mice (aged/Y). The gene expression profiles of aged/Y HSCs were reprogrammed to a large extent similar to those of young HSCs [130]. Another study showed that BM innervation by the SNS influenced the function of HSCs and that supplementation with a sympathomimetic (β3-AR agonist, BRL37344) significantly rejuvenated the in vivo functions of aged HSCs in old mice [109]. Furthermore, chronic treatment of progeroid mice with BRL37344 decreased premature myeloid and HSC expansion and restored the proximal association of HSCs to megakaryocytes [113].

## Conclusions

In the present review, we summarize the hallmarks of HSC aging in self-renewal, differentiation bias, and implantation ability. On the one hand, HSC aging is driven by multiple cell-intrinsic factors, including gene expression alterations, signaling pathway activation/repression, and epigenetic regulation. We review recent HSC aging studies with high-throughput single-cell sequencing at both the transcriptomic and epigenomic levels and summarize the single-cell sequencing data on age-associated surface molecules. On the other hand, some cell-extrinsic factors, including HSC-surrounding cells (such as megakaryocytes, MSCs, macrophages, and neutrophils), β-adrenergic nerve signals, cytokines, and enzymes (including IL-6, IL-1B, and caspase-1), also affect HSC aging. Finally, we review some strategies that have been employed to rejuvenate aged HSCs based on the above intrinsic and extrinsic mechanisms, including prolonged fasting, gene expression regulation, pharmacological intervention, and changing the BM niche.

Classical knowledge about hematopoiesis, which is built on a system defined with cell surface markers, is rather restricted and has been challenged. Compared with bulk sequencing, single-cell RNA sequencing technologies allow the dissection of gene expression at single-cell resolution, which provide unprecedented insight into cellular heterogeneity and HSC aging mechanisms. With evolvement of single cell technologies, researchers can profile multiple epigenetic marks within the same single cell and do so in combination with transcriptional information. However, considering the limitations of transcript coverage, low capture efficiency, high costs, and restricted cell throughput facing current single-cell sequencing methods, future single-cell strategies should be designed to conduct full-length sequencing and achieve a balance between high-throughput analysis and sufficient sequencing depth. In the future, single cell technologies and other emerging new technologies will pave the way for manipulation of the transcriptome and epigenome to rejuvenate aged HSC.

Although the latest single-cell studies delineated the association between functional decline and molecular mechanism, these studies did not conclusively identify driving factors of HSC aging. Whether these alterations drive HSC aging or whether these alterations are only accompanied by HSC aging remains unknown. Therefore, gene editing experiments should be performed to determine the specific functions of each gene. In addition, while many studies have directly studied aged HSCs, few studies have examined the HSC niche or revealed the specific functions of each cell subset in the HSC niche during aging. Furthermore, most strategies to rejuvenate aged HSCs directly act on HSCs, and few studies have tried to affect the HSC niche. Therefore, future work should emphasize the mechanisms of the HSC niche during aging. Moreover, expanding long-term HSCs in vitro is still a challenge, and the findings of HSC aging could be applied to this challenge [143–146]. For example, some rejuvenation molecules might be added to expand HSCs. Finally, the declining immune ability of the elderly population might be associated with aged hematopoietic cell dysfunction [147, 148]. These problems may be effectively overcome once HSC aging mechanisms are fully revealed and rejuvenation strategies are optimized.

## Data Availability

The material supporting the conclusion of this review has been included within the article.

## Abbreviations

HSCs: Hematopoietic stem cells
ROS: Reactive oxygen species
mTOR: Mammalian target of rapamycin
MAPK: Mitogen-activated protein kinase
LT-HSCs: Long-term HSCs
BM: Bone marrow
ST-HSCs: Short-term HSCs
MPP: Multipotent progenitors
HSPCs: Hematopoietic stem and progenitor cells
DDR: DNA damage response
NER: Nucleotide excision repair
NHEJ: Non-homologous end-joining
JAK/STAT: Janus kinase and signal transducer and activator of transcription
NF-κB: Nuclear factor-κB
TGF-β: Transforming growth factor-β
UPR^mt^: Mitochondrial unfolded protein response
TXNIP: Thioredoxin-interacting protein
FoxO: Forkhead O
5-mC: 5-Methylcytosine
5-hmC: 5-Hydroxymethylcytosine
scATAC-seq: Single-cell assay for transposase-accessible chromatin using sequencing
EpiTOF: Epigenetic landscape profiling using cytometry by time-of-flight
SNS: Sympathetic nervous system
ADRβ3: Adrenoreceptor β3
IL-6: Interleukin-6
IL-1B: Interleukin-1B
MSCs: Mesenchymal stromal cell
IGF-1: Insulin-like growth factor-1
